# On the Nature of Fear of Falling in Parkinson’s Disease

**DOI:** 10.3233/BEN-2011-0330

**Published:** 2011-08-29

**Authors:** S. Rahman, H. J. Griffin, N. P. Quinn, M. Jahanshahi

**Affiliations:** ^1^Sobell Department of Motor Neuroscience and Movement DisordersInstitute of Neurology and the National Hospital for Neurology and NeurosurgeryUniversity College LondonQueen SquareWC1N 3BGLondon; ^2^CognitivePerceptual and Brain SciencesUniversity College London26 Bedford WayWC1H 0APLondon

**Keywords:** Fear of falling, Parkinson's disease, mobility, quality of life, falls

## Abstract

In the elderly, fear of falling (FoF) can lead to activity restriction and affect quality of life (QoL). Our aim was to identify the characteristics of FoF in Parkinson's disease and assess its impact on QoL. We assessed FoF in 130 patients with Parkinson’s disease (PD) on scales measuring perceived self-efficacy in performing a range of activities (FES), perceived consequences of falling (CoF), and activity avoidance (SAFFE). A significant difference was found in FoF between PD patients who had previously fallen and those who had not and between frequent and infrequent fallers. Patient-rated disability significantly influenced FoF. Difficulty in rising from a chair, difficulty turning, start hesitation, festination, loss of balance, and shuffling were the specific mobility problems which were associated with greater FoF in PD. Disability was the main predictor of FoF, additionally depression predicted perceived consequences of falling, while anxiety predicted activity avoidance. The FoF measures explained 65% of the variance of QoL in PD, highlighting the clinical importance of FoF. These results have implications for the clinical management of FoF in PD.

## Abbreviations

| | |
|---|---|
| ADL | Activities of daily living |
| CoF | Consequences of falling |
| FES | Falls Efficacy Scale |
| FoF | Fear of falling |
| H&Y | Hoehn and Yahr stage |
| PD | Parkinson's disease |
| QoL | Quality of life |
| S&E | Schwab and England disability rating |
| SAFFE | Survey of Activities and Fear of Falling in the Elderly |

## 1. Introduction

In elderly populations, fear of falling (FoF) is a serious problem over and above falling itself [1–3]. FoF is associated with poorer health, reduced mobility and social activity, greater frailty, balance and gait problems, modified postural control, psychological distress in the form of anxiety and depression, poorer quality of life (QoL), and increased likelihood of nursing home admission [1–10]. FoF has been linked to subsequent falls [10–12] and vice versa, resulting in a spiraling incidence of both phenomena and subsequent functional decline [1,9,10,13].

Investigation of FoF in Parkinson's disease (PD) has been limited, despite the high risk of falls in PD [11, 14,15] and the proposal that the propensity to fall and an incapacitating fear of future falls lead to a restriction of daily activities [9,10,16–18], and a loss of independence, leaving patients socially isolated and reducing quality of life [19]. Activity restriction is in itself a risk factor for further falls and can lead to physical problems including muscle atrophy and poorer balance [3, 20,21]. PD patients have less confidence in performing activities of daily living (ADL) without falling than age-matched controls, and FoF is correlated with clinical ratings of balance and gait impairment [16,18,19, 22]. Such a relationship would be expected since falling itself has been linked to balance impairment [11,23].

[1]These authors contributed equally to this work.
*Corresponding author. Tel.: +44 20 3108 0033; Fax: +44 20 7419 1860; E-mail: m.jahanshahi@ion.ucl.ac.uk.

ISSN 0953-4180/11/$27.50 © 2011 – IOS Press and the authors. All rights reserved

The validity of general FoF measures has been shown in PD [18]; however, FoF in PD may also be influenced by specific mobility problems, such as festination and freezing of gait [24] in addition to previous falls. PD treatments such as medication [25,26] and deep brain stimulation (DBS) [27,28] also influence falls and FoF. Notwithstanding the importance of mobility problems, the contribution of non-motor symptoms such as anxiety and depression to FoF in PD is currently unclear. A strong association between affective variables and FoF has been found [29], as has an inter-relation between depression, anxiety, physical ability and FoF [30]. Recent data from Thomas et al. suggests that PD patients who fall frequently express *less* FoF than those who fall infrequently [24]. It is suggested that this may be due to previous experience of falls without adverse consequences and may be a valuable marker for patients who would benefit from special techniques to instill a more realistic perception of the risks and consequences of falls.

Single-item methodology for assessing FoF i.e.,"Are you afraid of falling?" is not adequately sensitive to different aspects of FoF, since FoF can be conceptualised in many ways: diminished perceived self-efficacy in performing a range of activities [9,30–32], avoidance of activity [9], loss of confidence [33] and as a specific expression of anxiety [9,34]. The question of how FoF is best measured remains open [4,35] and is of particular interest for the management of FoF in PD.

It has been previously reported that PD patients have greater FoF than similarly aged healthy controls [16]. FoF has also been noted as prevalent in progressive supranuclear palsy [36], degenerative cerebellar ataxia [37], multiple sclerosis [38] and other neurological conditions [11], but not extensively investigated. The sparsity of research on FoF, and the range of FoF measures used, makes comparison with previous studies of the healthy elderly population difficult (for a recent review of the use and validity of FoF questionnaires in healthy adult populations see [39]). Therefore, this study did not seek to explore the differences in FoF between PD patients and controls. Instead, our aim was a detailed investigation of the components and characteristics of FoF in a large sample of PD patients, with normative data available for comparison where appropriate. More specifically, the aims of the study were: (i) to undertake a multi-dimensional assessment of different aspects of FoF in PD and to identify the dominant features of FoF in terms of perceived self-efficacy, feared consequences of falling and range of activities avoided, (ii) to investigate the impact of disease stage, previous falls, fall frequency and the contribution of specific mobility problems as well as depression and anxiety to FoF in PD, and (iii) to identify the contribution of FoF to QoL in PD.

## 2. Methods

The study was approved by the Ethics Committee of the National Hospital for Neurology and Neurosurgery (NHNN) and the Institute of Neurology. Informed consent was obtained from all participants.

### 2.1. Sample

Patients who had recently attended PD clinics at the NHNN were invited to participate, 68% of whom responded. An advertisement was placed in the Parkinson's Disease Society Newsletter, to which 60 patients replied. The majority of questions could be answered by marking the chosen response and no writing was required. 130 patients (84 male) completed the questionnaires. Their mean age was 66.7 (SD 8.52). The mean duration of illness was 12.1 years (SD 7.94). The mean self-rated H&Y stage [40] for the sample was 2.63 (SD = 1.19).

### 2.2. Measures

#### 2.2.1. Stage of illness and disability

The self-administered measures of disease stage and of disability were modified versions of the Hoehn and Yahr (H&Y) scale [40]; with stage 1 indicating mild parkinsonism and stage 5 indicating the most severe disease, and the Schwab and England (S&E) scale [41]; with 10 indicating complete independence and 0 indicating complete dependence. Agreement between clinician and self-ratings of stage of illness and disability in PD has been found to be high [42].

#### 2.2.2. History of falls

Patients' history of falls was assessed as part of the gait and falls questionnaire [43]. Item 12: "How often do you fall?" Possible responses were 0 = Never, 1 = Very rarely – about once a year, 2 = Rarely – about once a month, 3 = Often – about once a week, 4 = Very often – once a day or more.

#### 2.2.3. PD mobility checklist

Patients indicated whether they experienced specific mobility symptoms (listed in Table 3). The terms *start*

*hesitancy, shuffling*, *freezing*, *festination*, *propulsion* and *retropulsion* were explained in simple terms. Each symptom was rated as 0 = absent, 1 = mild, 2 = moderate, 3 = severe, when on and off medication.

#### 2.2.4. *Measures of Fear of Falling (FoF)*

*Consequences of Falling (CoF):* Using the validated CoF scale [44], patients rated twelve statements of the form "I think that if I fall over ..." on 4-point scale (1 = disagree strongly, 2 = disagree, 3 = agree, 4 = agree strongly). The possible total score range was 12-48, with higher scores indicating greater concern about CoF. Two, six-item subscales (score range 6–24) were also calculated: damage to identity (CoF-DI), and loss of functional independence (CoF-LFI).

*Falls Efficacy Scale (FES):* Self-ratings of perceived efficacy in performing 10 non-hazardous activities of daily living without falling provided a validated measure of FoF [30]. The total FES score ranged from 10 (completely confident/no fear of falling) to 100 (not at all confident/high fear of falling).

*Survey of activities and fear of falling in the elderly (SAFFE):* Using the Modified Survey of Activities and Fear of Falling in the Elderly (SAFFE) [9,44], patients rated 17 activities on whether they would avoid them in case they fell (0 = never avoid, 1 = sometimes avoid, 2 = always avoid). The possible total score range was 0–34, with higher scores indicating higher avoidance of activities.

#### 2.2.5. *Measures of mood*

Self-reported depression and anxiety were assessed using the 21 item Beck Depression Inventory (BDI) [45] and Beck Anxiety Inventory (BAI) [46] questionnaires respectively. Both questionnaires had 21 items, scored 0–3, giving summary scores from 0 (no depression/anxiety) to 63 (greatest depression/anxiety).

#### 2.2.6. *Assessment of quality of life (QoL)*

QoL was assessed with the PDQ-39 [47], a PD-specific measure, from which the summary index (the PDQ39-SI) was derived, with a range of 0–100. Higher scores reflect poorer QoL.

### 2.3. *Statistics*

Data from the three FoF measures was inspected for normality and transformed if necessary. Effects of H&Y stage and previous falls on measures of FoF were assessed using ANOVA, correlations between FoF measures were analyzed using Pearson's correlation coefficient. Regression analysis was used to assess the proportion of variance of FoF measures explained by severity of illness and measures of mood and of QoL explained by FoF.

Table 1A
Consequence of Falling scale. Percentage of the sample who agreed or strongly agreed with each item. "I think that if I fall over...". Valid N ranged from 116 to 122

| | Percentage of sample who endorsed item |
|---|---|
| Damage to identity (CoF-DI) | |
| I will be embarrassed | 62.3% |
| I will be in pain | 53.8% |
| I will feel foolish | 51.5% |
| I will have difficulty getting up | 48.5% |
| I will lose my confidence | 43.8% |
| I will cause a nuisance | 33.8% |
| Loss of functional independence (CoF-LFI) | |
| I will not be able to cope alone | 35.4% |
| I will lose my independence | 31.5% |
| I will become disabled | 30.0% |
| I will be helpless | 26.9% |
| I cannot continue to be active | 25.4% |
| I will be severely injured | 20.0% |

## 3. Results

The mean CoF score was 26.9 (SD = 7.90, N = 110). CoF "damage to identity" (DI) subscore (mean = 14.6, SD = 4.29, N = 114) was significantly greater than the CoF "loss of functional independence" (LFI) subscore (mean = 12.5, SD = 4.34, N = 116; t(109) = 7.75, $p < 0.0005$, 2-tailed), reflecting the higher percentage of patients endorsing CoF-DI items (33.8%–62.3% per item) than CoF-LFI items (20.0%–35.4% per item; independent t-test, $p < 0.01$, 2-tailed) see Table 1A. Equivalent measurements of CoF in healthy controls are rare, but a significantly older group of healthy subjects (mean age = 80.7, SD 4.25; N = 166, $p < 0.0001$, 2-tailed) reported very similar perceived CoF (CoF-DI = 14.4, SD = 3.8; CoF-LFI = 12.4, SD = 4.0; independent t-tests both $p > 0.5$, 2-tailed) [44].

The mean FES score for the PD sample was 38.0 (SD = 24.8, N = 110). Studies of healthy subjects have reported greater self-efficacy (mean FES scores = 25.1, SD = 12.3; N = 56, $p < 0.001$, 2-tailed), i.e., lower FoF, despite sampling an older population (mean age = 78, $p < 0.0001$, 2-tailed) [30]. Our sample also reported low perceived self-efficacy relative to previous PD samples (mean FES score = 24.96) [24]. This difference may be due to longer disease duration (12.1 vs. 7.6 years, $p < 0.0001$, 2-tailed) and a higher percentage

Table 1B
Mean (SD) scores on the Falls Efficacy Scale. Valid N ranged from 121 to 125

| Activity | Confidence in performing activity without falling (1 = completely confident, 10 = not at all confident) |
|---|---|
| Simple shopping | 4.38 (3.28) |
| Light housekeeping | 4.30 (3.07) |
| Preparing meals not requiring carrying heavy or hot objects | 4.23 (3.15) |
| Getting dressed and undressed | 3.86 (2.53) |
| Taking a bath or a shower | 3.77 (2.66) |
| Walking around the house | 3.75 (2.59) |
| Getting in and out of bed | 3.67 (2.73) |
| Answering the door or telephone | 3.61 (2.64) |
| Reaching into cabinets or closets | 3.41 (2.56) |
| Getting in and out of a chair | 3.35 (2.45) |

Table 1C
Survey of Activities and Fear of Falling in the Elderly (SAFFE) Scale: Percentage of the sample who sometimes or always avoided each activity. Valid N ranged from 113 to 119

| Activity | Percentage of patients who avoided activity |
|---|---|
| Go out when it is slippery | 72.3% |
| Go to a place with crowds | 64.6% |
| Go out to a social event | 58.5% |
| Go for a walk | 53.8% |
| Travel by public transport | 52.3% |
| Go to the shops | 50.0% |
| Take a bath | 49.2% |
| Reach for something above head level | 48.5% |
| Walk half a mile | 43.8% |
| Clean your house | 41.5% |
| Bend down to get something | 40.8% |
| Visit a friend or relative | 39.2% |
| Go up and down stairs | 33.8% |
| Take a shower | 33.1% |
| Prepare simple meals | 26.2% |
| Go to the doctor or dentist | 18.5% |
| Walk around indoors | 16.2% |

of fallers in our sample (61.5% reporting previous falls vs. 41% reporting a fall in previous 6 months). Perceived self-efficacy was lowest for relatively complex activities and shopping, the only item that necessitates leaving the home, see Table 1B, a pattern seen in previous PD samples [18]. For further statistical analyses, FES scores were log-transformed to remove skew.

The mean SAFFE score was 10.2 (SD = 6.95, $N = 102$), previous measures of SAFFE in PD have produced similar mean values (8.2–9.2 after conversion for different scoring) [18]. The most frequently avoided activities involved venturing outside the home (Table 1C). However, excursions with a specific purpose such as visiting a relative or the doctor were less commonly avoided. Li et al. assessed 256 elderly subjects (mean age 77.5, SD = 5.0, $p < 0.0001$) on an 11-activity version of the SAFFE on both activity levels and FoF. These elderly controls reported similar avoidance of going out for a walk, especially when slippery.

Table 2
Mean (SD) values for fallers and non-fallers on measures of fear of falling CoF: Consequences of Falling Scale; CoF-DI: Consequences of Falling, damage to identity; CoF-LFI: Consequences of Falling, loss of functional independence; FES: Falls Efficacy Scale, SAFFE: Survey of Activities and Fear of Falling in the Elderly. Significance for FES is based on tests of logged FES scores

| Measure | Non-fallers | Fallers | p-value |
|---|---|---|---|
| Age | 64.5 (8.93) | 67.4 (8.16) | 0.081 |
| Years since PD onset | 8.56 (4.47) | 13.7 (8.47) | 0.001 |
| Hoehn and Yahr stage | 2.30 (1.04) | 2.77 (1.16) | 0.034 |
| CoF | 24.5 (7.71) | 28.4 (7.84) | 0.018 |
| CoF-DI | 13.6 (4.44) | 15.4 (4.12) | 0.042 |
| CoF-LFI | 11.1 (3.99) | 13.1 (4.38) | 0.022 |
| FES | 30.8 (25.1) | 40.4 (23.4) | 0.010 |
| SAFFE | 7.77 (6.19) | 11.5 (7.01) | 0.010 |

However, a large percentage of PD patients in our study reported avoiding going to places with crowds, including shops, whereas for elderly controls these are among the least feared activities [35].

Perceived consequences of falling were more severe in female PD patients than males (CoF: $F(1,108) = 5.14$, $p < 0.03$), specifically a greater fear of damage to identity (CoF-DI: $F(1,112) = 9.90$, $p = 0.002$). There were no other significant effects of gender on FoF.

CoF, (logged) FES and SAFFE scores were significantly correlated ($p < 0.0005$): CoF – FES $r = 0.396$, CoF – SAFFE $r = 0.660$, FES – SAFFE $r = 0.596$.

### 3.1. Previous falls and FoF

61.5% of patients reported previous falls, 30.0% had never fallen (8.5% did not respond to this item). 30.0% reported falling once a month or more and 13.8%, once a week or more. ANOVAs showed that fallers had greater FoF on all measures than non-fallers (all $p < 0.05$, see Table 2). Fallers tended to be older ($p < 0.09$) and had longer disease duration ($p = 0.001$) than non-fallers. As expected, fallers also had higher H&Y stage than non-fallers ($p < 0.04$), since higher H&Y

stages are defined by the presence of balance problems, of which falls would be the clearest sign. To determine whether the effect of falls on FoF is mediated by these differences, we repeated the analysis using ANCOVAs, with duration of illness and H&Y stage as covariates. Having controlled for these covariates, previous experience of falls did not significantly affect any measures of FoF (all $p > 0.05$).

Contrary to the findings of Thomas et al. FoF did not diminish with fall frequency (see Fig. 1) [24]. Comparison of infrequent fallers (approximately 1 fall per month or fewer) with frequent fallers (approximately 1 fall per week or more) revealed greater FoF in frequent fallers on all measures of FoF except CoF-DI (Bonferroni corrected criterion $p < 0.01$: CoF $p < 0.007$, CoF-DI $p > 0.1$, CoF-LFI $p < 0.002$, FES $p < 0.002$, SAFFE $p < 0.009$). However, inspection of the group of most frequent fallers revealed strikingly low minimum scores for CoF (minimum = 15) and SAFFE (minimum = 0).

### 3.2. *Effects of specific PD mobility symptoms on FoF scores*

Table 3 shows differences in FoF scores as a function of presence or absence of 11 PD mobility problems in the “on” state. After Bonferroni correction ($p < 0.0045$), significant effects on FoF scores were found for difficulty in rising from a chair, difficulty turning, start hesitation, festination, loss of balance, and shuffling. Perceived self-efficacy (measured by FES) appeared most sensitive to the presence of PD mobility symptoms.

### 3.3. *Predictors of FoF*

Multiple linear regressions were run with FoF measures as the dependent variables and H&Y, S&E, BDI and BAI as independent variables. For CoF these independent variables accounted for 43.5% of the variance; S&E disability rating ($\beta = -0.331$, $t = -3.67$, $p < 0.0005$), and BDI ($\beta = 0.362$, $t = 3.94$, $p < 0.0005$) were significant predictors of perceived consequences of falling (others $p > 0.19$). For FES, the independent variables accounted for 41.2% of the variance and only S&E disability rating ($\beta = -0.461$, $t = -4.97$, $p < 0.0005$) was a significant predictor of perceived self-efficacy (all others $p > 0.12$). For SAFFE, 50.7% of the variance was explained by the independent variables; S&E disability rating ($\beta = -0.504$, $t = -5.67$, $p < 0.0005$), and BAI ($\beta = 0.205$, $t = 2.27$, $p = 0.025$) were significant predictors of activity avoidance, as was BDI at trend level ($\beta = 0.165$, $t = 1.84$, $p = 0.069$).

### 3.4. *Effect of FoF on QoL*

CoF-DI, CoF-LFI, FES and SAFFE were entered as independent variables in a regression analysis with PDQ39-SI as the dependent variable. These measures of FoF collectively accounted for 65.2% of the variance in QoL. SAFFE ($\beta = 0.399$, $t = 3.44$, $p = 0.001$) and FES ($\beta = 0.240$, $t = 2.51$, $p = 0.015$) were significant predictors of QoL. A dissociation was found between CoF-DI which was not a significant predictor of QoL ($\beta = 0.047$, $t = 0.325$, $p > 0.7$) and CoF-LFI which approached significance ($\beta = 0.291$, $t = 1.99$, $p = 0.052$) as a predictor of QoL.

## 4. Discussion

This study aimed to assess multiple components of FoF in PD and their relationship with fall history, PD symptoms and measures of mood. FoF was influenced by disability (S&E), previous falls and the presence of specific PD mobility symptoms in addition to depression and anxiety. FoF, in turn, had a significant impact on QoL. These results also indicate of the value of the various FoF measures used to assess different aspects of FoF in PD patients, since intriguing differences emerged in how these scales related to measures of PD severity, PD symptoms and measures of mood.

Relative to previously published normative data from elderly healthy individuals our PD sample had greater fear of the consequences of falling and lower perceived self-efficacy. This is in agreement with previous studies, which found PD patients to report greater FoF [16]. Although the absence of a matched control group is a potential weakness of this study, our primary aim was the detailed investigation of the characteristics and components of FoF in PD and factors that contributed to it and how FoF, in turn, affected QoL. Therefore, we will focus on the discussion of these findings and the relevance of this information to clinical practice regarding the identification of FoF in PD and the strategies that should be used to prevent or ameliorate it.

That only moderate correlations appeared between different measures of FoF corroborates that they reflect different aspects of FoF [4]. A patient who believes they are likely to fall during a particular activity (as revealed by FES) does not necessarily avoid that activity, since they may consider the activity to be essential or the consequences of a fall to be minimal. Similarly, a patient who fears severe consequences of a fall (as measured by CoF) does not necessarily believe that a fall is

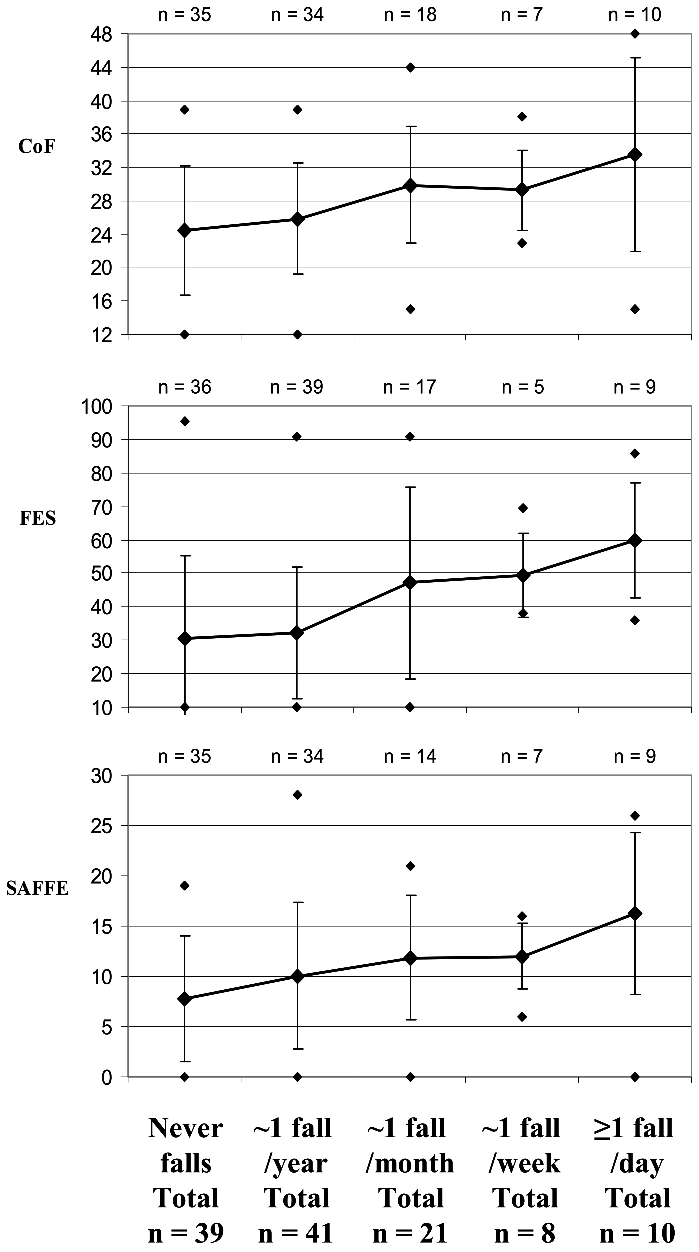

Fig. 1. Mean (± SD) fear of falling (FoF) values vs. frequency of falling. Higher values indicate greater FoF on all measures. Points above and below mean line indicate maximum and minimum values in each group. CoF: Consequences of Falling Scale; FES: Falls Efficacy Scale, SAFFE: Survey of Activities and Fear of Falling in the Elderly.

likely. Arguably, the SAFFE scale is the best measure of FoF since it measures actual activity avoidance due to FoF, the presence of which implies that (i) the patient believes that a fall is likely, (ii) they also believe that the fall will have undesirable consequences, and (iii) that these beliefs are strong enough to act on. That the SAFFE incorporates the crucial aspects of the FES and CoF measures in this way is supported by the relatively large correlations of FES with SAFFE, and CoF with SAFFE, compared to the correlation of FES with CoF.

Damage to identity was more commonly perceived as a likely consequence of falling than loss of functional independence, possibly reflecting existing limitations to functional independence due to PD. Fear of damage to identity was greater in women than men, corroborating previous findings [44], and similar to other

Table 3
Mean (SD) scores on the CoF, FES, and SAFFE according to the presence/absence of specific mobility problems. Comparisons that reach significance at the Bonferroni corrected criterion of $p < 0.0045$ are shown in bold, $p < 0.05$ are marked "*". Significance for FES is based on tests of log-transformed FES scores.

| Motor symptoms | | CoF | FES | SAFFE |
|---|---|---|---|---|
| Difficulty rising from a chair | Present | **28.4 (7.75)** | **41.8 (24.1)** | **12.0 (6.81)** |
| | Absent | **23.0 (6.38)** | **23.1 (20.6)** | **5.41 (4.67)** |
| Difficulty turning while walking | Present | **28.8 (7.67)** | **44.0 (23.4)** | **12.5 (6.66)** |
| | Absent | **24.2 (7.37)** | **27.6 (23.3)** | **7.10 (6.04)** |
| Start hesitation | Present | 28.9 (8.55)* | **46.0 (25.2)** | **12.1 (6.80)** |
| | Absent | 24.5 (5.99)* | **27.1 (19.6)** | **7.66 (5.98)** |
| Festination | Present | 28.5 (8.82) | **47.6 (24.7)** | **12.8 (7.23)** |
| | Absent | 26.0 (6.98) | **31.5 (22.7)** | **8.34 (5.88)** |
| Loss of balance | Present | 28.4 (7.97)* | **44.4 (24.2)** | 11.6 (7.13)* |
| | Absent | 23.8 (6.41)* | **24.9 (21.9)** | 7.81 (5.83)* |
| Shuffling | Present | 27.6 (8.59) | **43.0 (23.5)** | 11.6 (7.36)* |
| | Absent | 25.9 (6.13) | **27.6 (23.0)** | 8.03 (5.46)* |
| Freezing | Present | 28.2 (8.66) | 43.3 (25.6)* | 11.4 (7.15)* |
| | Absent | 25.3 (6.06) | 31.2 (23.1)* | 8.54 (5.99)* |
| Propulsion | Present | 28.2 (8.36) | 40.7 (24.2) | 11.2 (7.27) |
| | Absent | 25.4 (7.16) | 31.9 (23.3) | 8.67 (6.00) |
| Lack of arm swing | Present | 26.9 (7.70) | 38.2 (23.7) | 10.6 (7.12) |
| | Absent | 27.2 (8.86) | 35.8 (28.4) | 9.92 (6.93) |
| Retropulsion | Present | 30.7 (10.1) | 53.7 (25.2) | 11.5 (10.0) |
| | Absent | 26.9 (7.74) | 36.2 (23.8) | 10.3 (6.60) |
| Stooped posture | Present | 27.6 (8.10) | 38.6 (24.0) | 10.7 (7.37) |
| | Absent | 25.4 (7.46) | 32.7 (26.7) | 9.59 (5.84) |

CoF: Consequences of Falling Scale; FES: Falls Efficacy Scale; SAFFE: Survey of Activities and Fear of Falling in the Elderly.

gender differences found in FoF [1–3,11,18]. Healthy elderly patients have reported that the greatest fear is associated with "going out when it is slippery", "taking a walk for exercise", and "reaching for something over your head" [35]. Our results indicate that similar activities are avoided by PD patients and that confidence decreases with increasing task complexity, but that situations with large numbers of people e.g., crowds, social events and public transport, are also avoided as shown in previous PD samples [18]. Previous work by our group has shown that such situations trigger freezing in a large percentage of patients [48]. Therefore, such situations may be avoided because they increase the likelihood of falling through freezing.

That fallers had significantly more advanced, and a longer duration of, PD corroborates previous studies [14], as does greater FoF in those who had already fallen [13,18,25,49]. Given the link between FoF and postural instability in PD patients [16,19], (as in controls [6]) this emphasizes the importance of ensuring that patients do not get into a recursive pattern of FoF and falls. Balance is important for the safe performance of many activities required to remain independent [50, 51] and so loss of balance was, predictably, related to lower perceived self-efficacy. If, as Thomas et al. speculate, experience of falls without adverse consequences leads to low FoF in frequent fallers we could predict that they would report a high expectation of falling during tasks (FES) but minimal expected consequences of falling (CoF) and low activity avoidance (SAFFE) [24]. Our data do not show this pattern overall; FoF increased with fall frequency on all measures except CoF-DI, on which fall frequency had no effect. Given that Thomas et al. measured perceived self-efficacy (FES), their findings are surprising in that they suggest that increased fall frequency is associated with erroneously increased confidence in performing tasks without falling. We note that some very frequent fallers (one fall a day or more) in our sample display very low CoF and SAFFE scores. These patients may merit further investigation either because they have developed effective strategies for reducing the consequences of falls, which beneficially reduce FoF and activity avoidance, or because they paradoxically show little apprehension regarding the consequences of falls, which may in turn lead to less cautious behaviour.

The presence of start hesitation and freezing (at $p < 0.05$) were associated with greater FoF. Sudden and unpredictable gait blocks frequently result in falls [52]. Difficulty in walking prior to a fall is well recognized, and may be due not only to PD, but also related to comorbidities (such as osteoporosis) that are potentially treatable [52]. Festination had a significant effect on FoF, and it has been associated with frequent falls [53]. There was no significant difference in FoF according to presence/absence of stooped posture despite it being considered to increase the likelihood of forward falls, perhaps as the associated forward shift of the centre of gravity provides relative protection against a backward fall [54]. Difficulty rising from a chair and turning were also associated with greater FoF on all scales, identifying them as worthwhile targets for treatment by physiotherapy or other means.

An important finding in this study is that depression and anxiety had differential effects on FoF. Whereas self-rated disability was a predictor of FoF on all three scales, greater depression predicted perceived consequences of falling (CoF), while anxiety predicted activity avoidance (SAFFE). This finding reflects the specific nature of the different components of FoF and suggests that direct treatment of depression is important for improving QoL [55] and perceived consequences of falling in PD, while treating anxiety is necessary for reducing FoF-related activity avoidance.

The influence of FoF on QoL appears to be as important as the contributions of disability, depression and anxiety [55]. That the SAFFE was the main predictor of QoL was expected since it directly measures patients' activity avoidance as a consequence of FoF, and QoL is also partly determined by patients' ability to engage in diverse activities. High SAFFE scores may identify PD patients who would benefit from therapeutic intervention relating to their FoF and avoidance behaviour. The complementary information provided by the three scales should provide a comprehensive assessment of FoF, with some components being more amenable to intervention through cognitive behavioural techniques than others. Our results suggest that, alongside exercise regimes and fall-related multifactorial interventions identified as effective in reducing FoF in PD [56, 57] and the wider elderly population [58], medical or cognitive behavioural treatment of depression and anxiety should improve FoF in PD. In addition, cognitive behaviour therapy to modify negative cognitions relating to perceived self-efficacy and consequences of falling and to overcome avoidance of activities may prove of value in management of FoF in PD.

## Acknowledgements

We are grateful to all patients who participated.

This work was funded by the UK Parkinson's Disease Society.
